# (But­oxy­methyl­idene)di­methyl­aza­nium tetra­phenyl­borate aceto­nitrile monosolvate

**DOI:** 10.1107/S1600536814005674

**Published:** 2014-03-19

**Authors:** Ioannis Tiritiris, Stefan Saur, Willi Kantlehner

**Affiliations:** a Fakultät Chemie/Organische Chemie, Hochschule Aalen, Beethovenstrasse 1, D-73430 Aalen, Germany

## Abstract

In the title solvated salt, C_7_H_16_NO^+^·C_24_H_20_B^−^·C_2_H_3_N, the C—N bond lengths in the cation are 1.2831 (19), 1.467 (2) and 1.465 (2) Å, indicating double- and single-bond character, respectively. The C—O bond length of 1.2950 (18) Å shows a double-bond character, pointing towards charge delocalization within the NCO plane of the iminium ion. The two C atoms of the *n*-butyl group are disordered over the two sites, with refined occupancy ratios of 0.890 (5):0.110 (5) and 0.888 (4):0.112 (4). In the crystal, C—H⋯π inter­actions occur between the methine H atom, H atoms of the –N(CH_3_)_2_ and –CH_2_ groups of the cation, and two of the phenyl rings of the tetra­phenyl­borate anion. The latter inter­action forms an aromatic pocket in which the cation is embedded. Thus, a two-dimensional pattern is created in the *ac* plane.

## Related literature   

For the crystal structures of alkali metal tetra­phenyl­borates, see: Behrens *et al.* (2012 ▶). For the crystal structure of (meth­oxy­methyl­idene)di­methyl­aza­nium tetra­phenyl­borate aceto­nitrile monosolvate, see: Tiritiris *et al.* (2014 ▶).
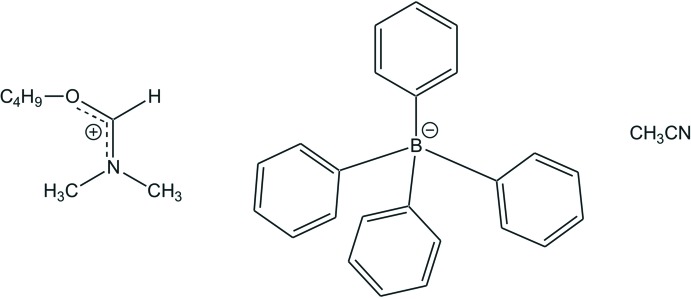


## Experimental   

### 

#### Crystal data  

C_7_H_16_NO^+^·C_24_H_20_B^−^·C_2_H_3_N
*M* *_r_* = 490.47
Monoclinic, 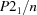

*a* = 11.2463 (4) Å
*b* = 17.7975 (9) Å
*c* = 14.6666 (7) Å
β = 100.821 (3)°
*V* = 2883.4 (2) Å^3^
*Z* = 4
Mo *K*α radiation
μ = 0.07 mm^−1^
*T* = 100 K
0.23 × 0.17 × 0.13 mm

#### Data collection  

Bruker Kappa APEXII DUO diffractometer
27626 measured reflections
7019 independent reflections
4718 reflections with *I* > 2σ(*I*)
*R*_int_ = 0.054

#### Refinement  

*R*[*F* ^2^ > 2σ(*F* ^2^)] = 0.049
*wR*(*F* ^2^) = 0.122
*S* = 1.01
7019 reflections
352 parameters
6 restraints
H atoms treated by a mixture of independent and constrained refinement
Δρ_max_ = 0.28 e Å^−3^
Δρ_min_ = −0.31 e Å^−3^

### 

Data collection: *APEX2* (Bruker, 2008 ▶); cell refinement: *SAINT* (Bruker, 2008 ▶); data reduction: *SAINT*; program(s) used to solve structure: *SHELXS97* (Sheldrick, 2008 ▶); program(s) used to refine structure: *SHELXL97* (Sheldrick, 2008 ▶); molecular graphics: *DIAMOND* (Brandenburg & Putz, 2005 ▶); software used to prepare material for publication: *SHELXL97*.

## Supplementary Material

Crystal structure: contains datablock(s) I, global. DOI: 10.1107/S1600536814005674/kp2466sup1.cif

Structure factors: contains datablock(s) I. DOI: 10.1107/S1600536814005674/kp2466Isup2.hkl

CCDC reference: 991457

Additional supporting information:  crystallographic information; 3D view; checkCIF report

## Figures and Tables

**Table 1:** Hydrogen-bond geometry (Å, °) *Cg*1 and *Cg*2 are the centroids of the C14–C19 and C8–C13 rings, respectively.

*D*—H⋯*A*	*D*—H	H⋯*A*	*D*⋯*A*	*D*—H⋯*A*
C3—H3⋯*Cg*1^i^	0.95 (2)	2.55 (2)	3.493 (2)	172 (2)
C2—H2*C*⋯*Cg*2^ii^	0.98	2.65	3.399 (2)	133
C5*A*—H5*B*⋯*Cg*2	0.99	2.78	3.621 (2)	144
C5*B*—H5*D*⋯*Cg*2	0.99	2.62	3.542 (2)	154

Symmetry codes: (i) 
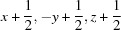
; (ii) 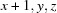.

**Fig. 1.**
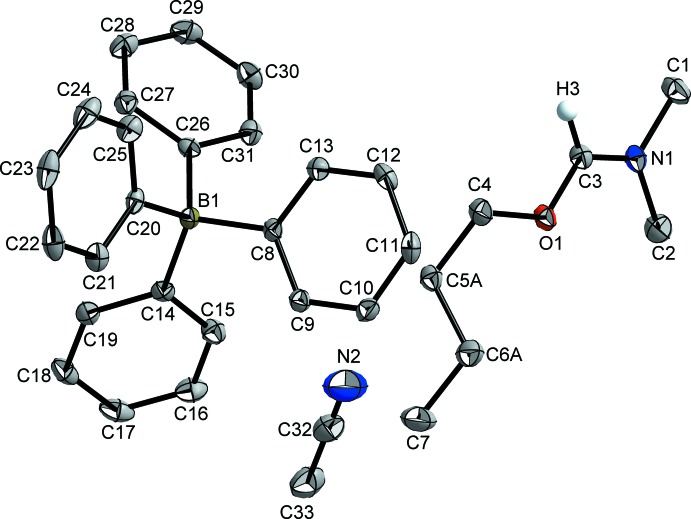
The structure of the title compound with displacement ellipsoids at the 50% probability level. All carbon bonded hydrogen atoms (except of H3) were omitted for the sake of clarity. Only the major orientation [pp = 0.888 (5)] of the n-butyl group is shown.

**Fig. 2.**
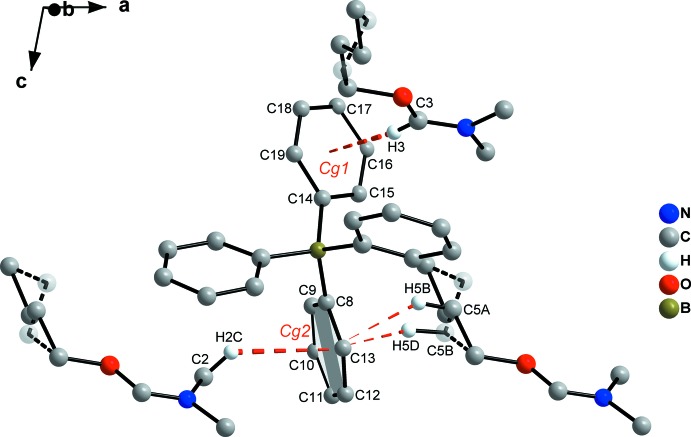
C—H···π interactions (red dashed lines) between the hydrogen atoms of the cation and the phenyl carbon atoms (centroids) of the tetraphenylborate ion. Both orientations of the n-butyl group are shown.

## supplementary crystallographic information

### 1. Comment

(Butoxymethylidene)dimethylazanium tetraphenylborate acetonitrile monosolvate is similar to the structurally known compound (methoxymethylidene)dimethylazanium tetraphenylborate acetonitrile monosolvate (Tiritiris *et al.*, 2014). According to the structure analysis, the C1–N1 bond length is 1.465 (2) Å, C2–N1 = 1.467 (2) Å and C3–N1 = 1.2831 (19) Å, showing single and double bond character, respectively. The C–N1–C angles are: 116.73 (12)° (C1–N1–C2), 121.54 (14)° (C1–N1–C3) and 121.65 (13)° (C3–N1–C2) indicating a nearly trigonal-planar surrounding of the nitrogen centre by the carbon atoms (Fig. 1). The C–O bond length of 1.2950 (18) Å reveals a double bond character. The positive charge is completely delocalized in the plane of the atoms N1, C3 and O1. The *n*-butyl group is disordered over the two sites with refined occupancies of 0.888 (5) and 0.112 (5). The bond lengths and angles in the tetraphenylborate anion are in good agreement with the data from the crystal structure analysis of the alkali metal tetraphenylborates (Behrens *et al.*, 2012). A strong C–H···π interaction between the hydrogen atom H3 of the cation and one phenyl ring (*Cg*1) of the tetraphenylborate anion is observed (Fig. 2). Slightly weaker C–H···π interactions between the hydrogen atoms of –N(CH_3_)_2_ and –CH_2_ groups and a second phenyl ring (*Cg*2) are also present (Fig. 2, Table 1). The hydrogen centroid distances are 2.55, 2.62, 2.65 and 2.78 Å (Tab. 1), respectively. The phenyl rings form aromatic pockets, in which the iminium ion is embedded. This leads to the formation of a two-dimensional supramolecular pattern in the *ac* plane. In contrast to the crystal structure of (methoxymethylidene)dimethylazanium tetraphenylborate acetonitrile monosolvate (Tiritiris *et al.*, 2014), the acetonitrile molecule is hardly involved in a C–H···N hydrogen bond system.

### 2. Experimental

The title compound was obtained by reacting of equimolar amounts of *N*,*N*-dimethylformamide with dimethyl sulfate at room temperature forming (methoxymethylidene)dimethylazanium methyl sulfate (I). One mol of (I) was heated with 2.2 mol n-butanol for eight hours at 313 K. The methanol formed was distilled off and (butoxymethylidene)dimethylazanium butyl sulfate (II) was obtained in nearly quantitative yield. 1.00 g (3.66 mmol) of crude (II) was dissolved in 20 ml acetonitrile and 1.25 g (3.66 mmol) of sodium tetraphenylborate in 20 ml acetonitrile was added. After stirring for one hour at room temperature, the precipitated sodium butyl sulfate was filtered off. The title compound crystallized from a saturated acetonitrile solution after several days at 273 K, forming colourless single crystals suitable for X-ray analysis.

Dimethyl sulfate is carcinogenic, mutagenic and highly poisonous. During the use appropriate precautions must be taken.

### 3. Refinement

The H atom bound to C3 was located in a difference Fourier map and was refined freely [C—H = 0.95 (2) Å]. The hydrogen atoms of the methyl groups were allowed to rotate with a fixed angle around the C–N, C–O and C–C bonds to best fit the experimental electron density, with *U*_iso_(H) set to 1.5*U*_eq_(C) and d(C—H) = 0.98 Å. The remaining H atoms were placed in calculated positions with d(C—H) = 0.99 Å (H atoms in CH_2_ groups) and (C—H) = 0.95 Å (H atoms in aromatic rings). They were included in the refinement in the riding model approximation, with *U*(H) set to 1.2 *U*_eq_(C).

Atoms C5 and C6 of the *n*-butyl group are disordered over two sites (C5A, C6A and C5B,C6B) with refined occupancies of 0.888 (5) and 0.112 (5). A free refinement of the anisotropic displacement parameters of the atoms C5B and C6B (minor moiety) was not possible, so an ISOR = 0.001 instruction for C6B was established, which solves this problem. Finally, the atoms C5B and C6B were restrained to have similar anisotropic displacement parameters. Nevertheless, it was not possible to prevent the detection of an A-alert in the checkcif utility. There is a large *U*_eq_(max)/*U*_eq_(min) ratio of the hydrogen atoms, caused by the large *U*_eq_ of the terminal methyl group hydrogen atoms and the small *U*_eq_ of the hydrogen atoms attached to the atoms C5B and C6B in the disordered *n*-butyl moiety.

### Crystal data

**Table:**

C_7_H_16_NO^+^·C_24_H_20_B^−^·C_2_H_3_N	*F*(000) = 1056
*M**_r_* = 490.47	*D*_x_ = 1.130 Mg m^−^^3^
Monoclinic, *P*2_1_/*n*	Mo *K*α radiation, λ = 0.71073 Å
Hall symbol: -P 2yn	Cell parameters from 27626 reflections
*a* = 11.2463 (4) Å	θ = 1.8–28.3°
*b* = 17.7975 (9) Å	µ = 0.07 mm^−^^1^
*c* = 14.6666 (7) Å	*T* = 100 K
β = 100.821 (3)°	Block, colourless
*V* = 2883.4 (2) Å^3^	0.23 × 0.17 × 0.13 mm
*Z* = 4	

### Data collection

**Table:**

Bruker Kappa APEXII DUO diffractometer	4718 reflections with *I* > 2σ(*I*)
Radiation source: fine-focus sealed tube	*R*_int_ = 0.054
Graphite monochromator	θ_max_ = 28.3°, θ_min_ = 1.8°
φ scans, and ω scans	*h* = −14→14
27626 measured reflections	*k* = −23→23
7019 independent reflections	*l* = −18→19

### Refinement

**Table:**

Refinement on *F*^2^	Primary atom site location: structure-invariant direct methods
Least-squares matrix: full	Secondary atom site location: difference Fourier map
*R*[*F*^2^ > 2σ(*F*^2^)] = 0.049	Hydrogen site location: inferred from neighbouring sites
*wR*(*F*^2^) = 0.122	H atoms treated by a mixture of independent and constrained refinement
*S* = 1.01	*w* = 1/[σ^2^(*F*_o_^2^) + (0.0513*P*)^2^ + 0.6909*P*] where *P* = (*F*_o_^2^ + 2*F*_c_^2^)/3
7019 reflections	(Δ/σ)_max_ < 0.001
352 parameters	Δρ_max_ = 0.28 e Å^−^^3^
6 restraints	Δρ_min_ = −0.31 e Å^−^^3^

### Special details

**Table:**

Geometry. All e.s.d.'s (except the e.s.d. in the dihedral angle between two l.s. planes) are estimated using the full covariance matrix. The cell e.s.d.'s are taken into account individually in the estimation of e.s.d.'s in distances, angles and torsion angles; correlations between e.s.d.'s in cell parameters are only used when they are defined by crystal symmetry. An approximate (isotropic) treatment of cell e.s.d.'s is used for estimating e.s.d.'s involving l.s. planes.
Refinement. Refinement of *F*^2^ against ALL reflections. The weighted *R*-factor *wR* and goodness of fit *S* are based on *F*^2^, conventional *R*-factors *R* are based on *F*, with *F* set to zero for negative *F*^2^. The threshold expression of *F*^2^ > σ(*F*^2^) is used only for calculating *R*-factors(gt) *etc*. and is not relevant to the choice of reflections for refinement. *R*-factors based on *F*^2^ are statistically about twice as large as those based on *F*, and *R*- factors based on ALL data will be even larger.

### Fractional atomic coordinates and isotropic or equivalent isotropic displacement parameters (Å^2^)

**Table:**

	*x*	*y*	*z*	*U*_iso_*/*U*_eq_	Occ. (<1)
N1	1.05819 (11)	0.19695 (7)	1.00987 (8)	0.0190 (3)	
C1	1.14020 (14)	0.25442 (10)	1.05776 (12)	0.0283 (4)	
H1A	1.0929	0.2939	1.0810	0.042*	
H1B	1.1870	0.2763	1.0144	0.042*	
H1C	1.1955	0.2315	1.1099	0.042*	
C2	1.11443 (15)	0.13197 (10)	0.97325 (12)	0.0275 (4)	
H2A	1.0513	0.0972	0.9437	0.041*	
H2B	1.1680	0.1063	1.0242	0.041*	
H2C	1.1616	0.1490	0.9274	0.041*	
C3	0.94283 (13)	0.20482 (9)	0.99683 (10)	0.0189 (3)	
H3	0.9082 (15)	0.2476 (10)	1.0209 (11)	0.025 (4)*	
O1	0.87394 (9)	0.15411 (6)	0.95062 (7)	0.0196 (2)	
C4	0.74205 (13)	0.16696 (9)	0.93430 (11)	0.0218 (3)	
H4A	0.7242	0.2209	0.9218	0.026*	
H4B	0.7093	0.1521	0.9898	0.026*	
C5A	0.68533 (15)	0.12077 (10)	0.85260 (13)	0.0197 (5)	0.890 (5)
H5A	0.7175	0.1374	0.7976	0.024*	0.890 (5)
H5B	0.5969	0.1300	0.8398	0.024*	0.890 (5)
C6A	0.70789 (17)	0.03642 (10)	0.86623 (14)	0.0277 (5)	0.888 (4)
H6A	0.7962	0.0271	0.8814	0.033*	0.888 (4)
H6B	0.6722	0.0190	0.9193	0.033*	0.888 (4)
C5B	0.6903 (11)	0.0909 (7)	0.8972 (10)	0.009 (3)	0.110 (5)
H5C	0.7235	0.0513	0.9422	0.011*	0.110 (5)
H5D	0.6014	0.0917	0.8928	0.011*	0.110 (5)
C6B	0.7178 (10)	0.0710 (6)	0.8041 (8)	0.009 (3)	0.112 (4)
H6C	0.8062	0.0669	0.8069	0.011*	0.112 (4)
H6D	0.6845	0.1091	0.7570	0.011*	0.112 (4)
C7	0.65349 (17)	−0.00822 (11)	0.78020 (14)	0.0410 (5)	
H7A	0.6697	−0.0618	0.7917	0.062*	
H7B	0.6898	0.0082	0.7278	0.062*	
H7C	0.5659	0.0002	0.7656	0.062*	
B1	0.29162 (15)	0.22427 (9)	0.73749 (11)	0.0157 (3)	
C8	0.35342 (12)	0.18276 (8)	0.83517 (10)	0.0145 (3)	
C9	0.35346 (13)	0.10426 (8)	0.84668 (10)	0.0173 (3)	
H9A	0.3228	0.0737	0.7945	0.021*	
C10	0.39659 (13)	0.06971 (9)	0.93151 (11)	0.0208 (3)	
H10A	0.3966	0.0165	0.9361	0.025*	
C11	0.43964 (13)	0.11303 (9)	1.00947 (11)	0.0223 (3)	
H11A	0.4693	0.0897	1.0675	0.027*	
C12	0.43886 (13)	0.19056 (9)	1.00171 (10)	0.0202 (3)	
H12A	0.4663	0.2207	1.0549	0.024*	
C13	0.39780 (13)	0.22429 (8)	0.91588 (10)	0.0179 (3)	
H13A	0.3999	0.2775	0.9117	0.022*	
C14	0.29953 (13)	0.17201 (8)	0.64632 (10)	0.0162 (3)	
C15	0.40218 (14)	0.12851 (8)	0.64120 (11)	0.0209 (3)	
H15A	0.4655	0.1264	0.6941	0.025*	
C16	0.41546 (15)	0.08837 (9)	0.56222 (11)	0.0259 (4)	
H16A	0.4866	0.0596	0.5622	0.031*	
C17	0.32541 (15)	0.09030 (9)	0.48393 (11)	0.0269 (4)	
H17A	0.3336	0.0627	0.4300	0.032*	
C18	0.22291 (15)	0.13319 (9)	0.48557 (10)	0.0242 (3)	
H18A	0.1604	0.1354	0.4322	0.029*	
C19	0.21100 (14)	0.17302 (8)	0.56495 (10)	0.0188 (3)	
H19A	0.1400	0.2021	0.5641	0.023*	
C20	0.15186 (13)	0.24097 (8)	0.75026 (9)	0.0168 (3)	
C21	0.05601 (13)	0.19093 (9)	0.72214 (10)	0.0207 (3)	
H21A	0.0714	0.1458	0.6919	0.025*	
C22	−0.06111 (14)	0.20452 (10)	0.73668 (11)	0.0263 (4)	
H22A	−0.1240	0.1695	0.7153	0.032*	
C23	−0.08547 (14)	0.26923 (10)	0.78234 (11)	0.0289 (4)	
H23A	−0.1653	0.2793	0.7916	0.035*	
C24	0.00722 (15)	0.31904 (10)	0.81425 (11)	0.0281 (4)	
H24A	−0.0082	0.3629	0.8470	0.034*	
C25	0.12320 (14)	0.30481 (9)	0.79832 (11)	0.0222 (3)	
H25A	0.1857	0.3397	0.8209	0.027*	
C26	0.36367 (13)	0.30231 (8)	0.72107 (10)	0.0160 (3)	
C27	0.30508 (14)	0.36477 (8)	0.67425 (10)	0.0194 (3)	
H27A	0.2197	0.3630	0.6544	0.023*	
C28	0.36669 (15)	0.42920 (9)	0.65570 (11)	0.0247 (4)	
H28A	0.3233	0.4700	0.6235	0.030*	
C29	0.49117 (15)	0.43391 (9)	0.68403 (11)	0.0260 (4)	
H29A	0.5335	0.4780	0.6723	0.031*	
C30	0.55292 (14)	0.37329 (9)	0.72968 (11)	0.0235 (3)	
H30A	0.6383	0.3756	0.7492	0.028*	
C31	0.49002 (14)	0.30897 (8)	0.74708 (10)	0.0192 (3)	
H31A	0.5345	0.2679	0.7778	0.023*	
N2	0.8185 (2)	0.08990 (10)	0.54785 (13)	0.0595 (5)	
C32	0.83893 (19)	0.06303 (10)	0.48185 (14)	0.0384 (5)	
C33	0.86399 (19)	0.02888 (10)	0.39784 (13)	0.0388 (5)	
H33A	0.7999	−0.0072	0.3738	0.058*	
H33B	0.9420	0.0028	0.4116	0.058*	
H33C	0.8670	0.0680	0.3512	0.058*	

### Atomic displacement parameters (Å^2^)

**Table:**

	*U*^11^	*U*^22^	*U*^33^	*U*^12^	*U*^13^	*U*^23^
N1	0.0166 (6)	0.0248 (7)	0.0156 (6)	−0.0002 (5)	0.0029 (5)	0.0000 (5)
C1	0.0179 (8)	0.0332 (9)	0.0324 (9)	−0.0040 (7)	0.0015 (7)	−0.0103 (7)
C2	0.0190 (8)	0.0326 (9)	0.0315 (9)	0.0040 (7)	0.0065 (7)	−0.0064 (7)
C3	0.0166 (7)	0.0227 (8)	0.0172 (7)	0.0002 (6)	0.0026 (6)	−0.0008 (6)
O1	0.0124 (5)	0.0246 (6)	0.0212 (6)	0.0001 (4)	0.0018 (4)	−0.0037 (4)
C4	0.0116 (7)	0.0268 (8)	0.0270 (8)	0.0014 (6)	0.0037 (6)	−0.0049 (6)
C5A	0.0159 (8)	0.0226 (10)	0.0202 (10)	0.0005 (7)	0.0025 (7)	−0.0003 (8)
C6A	0.0216 (10)	0.0234 (10)	0.0371 (12)	0.0006 (7)	0.0030 (8)	−0.0020 (8)
C5B	0.009 (3)	0.009 (3)	0.009 (3)	0.0001 (10)	0.0018 (11)	0.0003 (10)
C6B	0.009 (3)	0.009 (3)	0.009 (3)	0.0001 (10)	0.0018 (11)	0.0003 (10)
C7	0.0358 (11)	0.0293 (10)	0.0552 (13)	0.0015 (8)	0.0014 (9)	−0.0192 (9)
B1	0.0142 (8)	0.0163 (8)	0.0163 (8)	0.0005 (6)	0.0019 (6)	−0.0007 (6)
C8	0.0095 (6)	0.0175 (7)	0.0168 (7)	0.0009 (5)	0.0032 (5)	−0.0007 (5)
C9	0.0142 (7)	0.0185 (7)	0.0188 (7)	0.0006 (6)	0.0022 (6)	−0.0023 (6)
C10	0.0179 (7)	0.0181 (7)	0.0262 (8)	0.0036 (6)	0.0032 (6)	0.0020 (6)
C11	0.0154 (7)	0.0313 (9)	0.0190 (8)	0.0024 (6)	−0.0004 (6)	0.0061 (6)
C12	0.0148 (7)	0.0292 (8)	0.0162 (7)	−0.0036 (6)	0.0015 (6)	−0.0043 (6)
C13	0.0152 (7)	0.0180 (7)	0.0210 (8)	−0.0003 (6)	0.0045 (6)	−0.0015 (6)
C14	0.0167 (7)	0.0151 (7)	0.0172 (7)	−0.0030 (6)	0.0043 (6)	0.0016 (6)
C15	0.0202 (8)	0.0230 (8)	0.0196 (8)	−0.0002 (6)	0.0041 (6)	−0.0015 (6)
C16	0.0294 (9)	0.0238 (8)	0.0274 (9)	0.0022 (7)	0.0131 (7)	−0.0040 (7)
C17	0.0390 (10)	0.0238 (8)	0.0206 (8)	−0.0076 (7)	0.0130 (7)	−0.0066 (7)
C18	0.0294 (9)	0.0273 (8)	0.0149 (8)	−0.0091 (7)	0.0015 (6)	−0.0003 (6)
C19	0.0192 (7)	0.0189 (7)	0.0181 (7)	−0.0029 (6)	0.0031 (6)	0.0023 (6)
C20	0.0168 (7)	0.0203 (7)	0.0132 (7)	0.0030 (6)	0.0028 (6)	0.0041 (6)
C21	0.0181 (8)	0.0252 (8)	0.0191 (8)	−0.0006 (6)	0.0042 (6)	0.0040 (6)
C22	0.0163 (8)	0.0407 (10)	0.0214 (8)	−0.0030 (7)	0.0024 (6)	0.0082 (7)
C23	0.0167 (8)	0.0460 (11)	0.0259 (9)	0.0091 (7)	0.0090 (7)	0.0134 (8)
C24	0.0295 (9)	0.0312 (9)	0.0265 (9)	0.0127 (7)	0.0127 (7)	0.0056 (7)
C25	0.0209 (8)	0.0237 (8)	0.0230 (8)	0.0015 (6)	0.0066 (6)	0.0020 (6)
C26	0.0178 (7)	0.0171 (7)	0.0137 (7)	−0.0005 (6)	0.0041 (6)	−0.0026 (5)
C27	0.0184 (7)	0.0211 (8)	0.0187 (8)	0.0016 (6)	0.0035 (6)	−0.0006 (6)
C28	0.0296 (9)	0.0200 (8)	0.0255 (9)	0.0026 (7)	0.0074 (7)	0.0039 (6)
C29	0.0314 (9)	0.0212 (8)	0.0269 (9)	−0.0086 (7)	0.0095 (7)	0.0007 (7)
C30	0.0196 (8)	0.0285 (9)	0.0227 (8)	−0.0052 (7)	0.0045 (6)	−0.0007 (7)
C31	0.0187 (7)	0.0212 (8)	0.0179 (7)	0.0011 (6)	0.0037 (6)	0.0008 (6)
N2	0.0909 (16)	0.0368 (10)	0.0494 (11)	−0.0001 (10)	0.0097 (11)	−0.0072 (9)
C32	0.0497 (12)	0.0227 (9)	0.0394 (11)	−0.0011 (8)	−0.0005 (9)	0.0051 (8)
C33	0.0516 (12)	0.0290 (10)	0.0366 (11)	0.0036 (9)	0.0107 (9)	0.0084 (8)

### Geometric parameters (Å, º)

**Table:**

N1—C3	1.2831 (19)	C11—H11A	0.9500
N1—C1	1.465 (2)	C12—C13	1.393 (2)
N1—C2	1.467 (2)	C12—H12A	0.9500
C1—H1A	0.9800	C13—H13A	0.9500
C1—H1B	0.9800	C14—C19	1.403 (2)
C1—H1C	0.9800	C14—C15	1.404 (2)
C2—H2A	0.9800	C15—C16	1.393 (2)
C2—H2B	0.9800	C15—H15A	0.9500
C2—H2C	0.9800	C16—C17	1.382 (2)
C3—O1	1.2950 (18)	C16—H16A	0.9500
C3—H3	0.954 (18)	C17—C18	1.387 (2)
O1—C4	1.4752 (17)	C17—H17A	0.9500
C4—C5A	1.494 (2)	C18—C19	1.391 (2)
C4—C5B	1.531 (12)	C18—H18A	0.9500
C4—H4A	0.9900	C19—H19A	0.9500
C4—H4B	0.9900	C20—C21	1.399 (2)
C5A—C6A	1.530 (3)	C20—C25	1.406 (2)
C5A—H5A	0.9900	C21—C22	1.394 (2)
C5A—H5B	0.9900	C21—H21A	0.9500
C6A—C7	1.520 (3)	C22—C23	1.385 (2)
C6A—H6A	0.9900	C22—H22A	0.9500
C6A—H6B	0.9900	C23—C24	1.381 (2)
C5B—C6B	1.486 (13)	C23—H23A	0.9500
C5B—H5C	0.9900	C24—C25	1.391 (2)
C5B—H5D	0.9900	C24—H24A	0.9500
C6B—C7	1.5972	C25—H25A	0.9500
C6B—H6C	0.9900	C26—C27	1.404 (2)
C6B—H6D	0.9900	C26—C31	1.406 (2)
C7—H7A	0.9800	C27—C28	1.393 (2)
C7—H7B	0.9800	C27—H27A	0.9500
C7—H7C	0.9800	C28—C29	1.386 (2)
B1—C20	1.645 (2)	C28—H28A	0.9500
B1—C14	1.645 (2)	C29—C30	1.386 (2)
B1—C8	1.645 (2)	C29—H29A	0.9500
B1—C26	1.648 (2)	C30—C31	1.394 (2)
C8—C13	1.405 (2)	C30—H30A	0.9500
C8—C9	1.407 (2)	C31—H31A	0.9500
C9—C10	1.391 (2)	N2—C32	1.141 (3)
C9—H9A	0.9500	C32—C33	1.448 (3)
C10—C11	1.389 (2)	C33—H33A	0.9800
C10—H10A	0.9500	C33—H33B	0.9800
C11—C12	1.384 (2)	C33—H33C	0.9800
			
C3—N1—C1	121.54 (14)	C11—C10—C9	120.02 (14)
C3—N1—C2	121.65 (13)	C11—C10—H10A	120.0
C1—N1—C2	116.73 (12)	C9—C10—H10A	120.0
N1—C1—H1A	109.5	C12—C11—C10	119.33 (14)
N1—C1—H1B	109.5	C12—C11—H11A	120.3
H1A—C1—H1B	109.5	C10—C11—H11A	120.3
N1—C1—H1C	109.5	C11—C12—C13	119.99 (14)
H1A—C1—H1C	109.5	C11—C12—H12A	120.0
H1B—C1—H1C	109.5	C13—C12—H12A	120.0
N1—C2—H2A	109.5	C12—C13—C8	122.66 (14)
N1—C2—H2B	109.5	C12—C13—H13A	118.7
H2A—C2—H2B	109.5	C8—C13—H13A	118.7
N1—C2—H2C	109.5	C19—C14—C15	114.89 (14)
H2A—C2—H2C	109.5	C19—C14—B1	123.09 (13)
H2B—C2—H2C	109.5	C15—C14—B1	121.73 (13)
N1—C3—O1	119.39 (14)	C16—C15—C14	123.03 (14)
N1—C3—H3	120.3 (10)	C16—C15—H15A	118.5
O1—C3—H3	120.3 (10)	C14—C15—H15A	118.5
C3—O1—C4	117.47 (12)	C17—C16—C15	120.15 (15)
O1—C4—C5A	108.05 (12)	C17—C16—H16A	119.9
O1—C4—C5B	102.9 (5)	C15—C16—H16A	119.9
O1—C4—H4A	110.1	C16—C17—C18	118.77 (15)
C5A—C4—H4A	110.1	C16—C17—H17A	120.6
C5B—C4—H4A	137.7	C18—C17—H17A	120.6
O1—C4—H4B	110.1	C17—C18—C19	120.41 (14)
C5A—C4—H4B	110.1	C17—C18—H18A	119.8
C5B—C4—H4B	83.2	C19—C18—H18A	119.8
H4A—C4—H4B	108.4	C18—C19—C14	122.74 (15)
C4—C5A—C6A	113.56 (16)	C18—C19—H19A	118.6
C4—C5A—H5A	108.9	C14—C19—H19A	118.6
C6A—C5A—H5A	108.9	C21—C20—C25	115.19 (14)
C4—C5A—H5B	108.9	C21—C20—B1	123.45 (13)
C6A—C5A—H5B	108.9	C25—C20—B1	121.14 (13)
H5A—C5A—H5B	107.7	C22—C21—C20	122.83 (15)
C7—C6A—C5A	111.92 (16)	C22—C21—H21A	118.6
C7—C6A—H6A	109.2	C20—C21—H21A	118.6
C5A—C6A—H6A	109.2	C23—C22—C21	119.75 (16)
C7—C6A—H6B	109.2	C23—C22—H22A	120.1
C5A—C6A—H6B	109.2	C21—C22—H22A	120.1
H6A—C6A—H6B	107.9	C24—C23—C22	119.48 (15)
C6B—C5B—C4	113.8 (9)	C24—C23—H23A	120.3
C6B—C5B—H5C	108.8	C22—C23—H23A	120.3
C4—C5B—H5C	108.8	C23—C24—C25	119.87 (16)
C6B—C5B—H5D	108.8	C23—C24—H24A	120.1
C4—C5B—H5D	108.8	C25—C24—H24A	120.1
H5C—C5B—H5D	107.7	C24—C25—C20	122.81 (15)
C5B—C6B—C7	104.7 (5)	C24—C25—H25A	118.6
C5B—C6B—H6C	110.8	C20—C25—H25A	118.6
C7—C6B—H6C	110.8	C27—C26—C31	115.07 (13)
C5B—C6B—H6D	110.8	C27—C26—B1	122.69 (13)
C7—C6B—H6D	110.8	C31—C26—B1	122.08 (13)
H6C—C6B—H6D	108.9	C28—C27—C26	122.85 (14)
C6A—C7—H7A	109.5	C28—C27—H27A	118.6
C6B—C7—H7A	139.2	C26—C27—H27A	118.6
C6A—C7—H7B	109.5	C29—C28—C27	120.17 (15)
C6B—C7—H7B	71.2	C29—C28—H28A	119.9
H7A—C7—H7B	109.5	C27—C28—H28A	119.9
C6A—C7—H7C	109.5	C30—C29—C28	118.96 (15)
C6B—C7—H7C	108.2	C30—C29—H29A	120.5
H7A—C7—H7C	109.5	C28—C29—H29A	120.5
H7B—C7—H7C	109.5	C29—C30—C31	120.15 (15)
C20—B1—C14	113.17 (12)	C29—C30—H30A	119.9
C20—B1—C8	103.80 (11)	C31—C30—H30A	119.9
C14—B1—C8	112.05 (12)	C30—C31—C26	122.78 (14)
C20—B1—C26	111.78 (12)	C30—C31—H31A	118.6
C14—B1—C26	104.75 (11)	C26—C31—H31A	118.6
C8—B1—C26	111.51 (11)	N2—C32—C33	179.6 (2)
C13—C8—C9	115.41 (13)	C32—C33—H33A	109.5
C13—C8—B1	121.50 (12)	C32—C33—H33B	109.5
C9—C8—B1	122.73 (12)	H33A—C33—H33B	109.5
C10—C9—C8	122.57 (13)	C32—C33—H33C	109.5
C10—C9—H9A	118.7	H33A—C33—H33C	109.5
C8—C9—H9A	118.7	H33B—C33—H33C	109.5
			
C1—N1—C3—O1	177.02 (13)	C14—C15—C16—C17	0.2 (2)
C2—N1—C3—O1	0.4 (2)	C15—C16—C17—C18	0.5 (2)
N1—C3—O1—C4	−177.73 (13)	C16—C17—C18—C19	−0.4 (2)
C3—O1—C4—C5A	158.30 (14)	C17—C18—C19—C14	−0.3 (2)
C3—O1—C4—C5B	−168.8 (6)	C15—C14—C19—C18	0.9 (2)
O1—C4—C5A—C6A	60.11 (19)	B1—C14—C19—C18	174.79 (13)
C5B—C4—C5A—C6A	−25.5 (8)	C14—B1—C20—C21	30.61 (19)
C4—C5A—C6A—C7	−177.50 (15)	C8—B1—C20—C21	−91.10 (16)
O1—C4—C5B—C6B	−66.3 (9)	C26—B1—C20—C21	148.59 (13)
C5A—C4—C5B—C6B	37.1 (6)	C14—B1—C20—C25	−155.05 (13)
C4—C5B—C6B—C7	−178.7 (6)	C8—B1—C20—C25	83.24 (16)
C5A—C6A—C7—C6B	35.93 (11)	C26—B1—C20—C25	−37.07 (18)
C5B—C6B—C7—C6A	−27.7 (5)	C25—C20—C21—C22	2.8 (2)
C20—B1—C8—C13	−81.68 (15)	B1—C20—C21—C22	177.43 (14)
C14—B1—C8—C13	155.86 (13)	C20—C21—C22—C23	−1.3 (2)
C26—B1—C8—C13	38.81 (18)	C21—C22—C23—C24	−1.0 (2)
C20—B1—C8—C9	91.09 (15)	C22—C23—C24—C25	1.6 (2)
C14—B1—C8—C9	−31.36 (18)	C23—C24—C25—C20	0.1 (2)
C26—B1—C8—C9	−148.42 (13)	C21—C20—C25—C24	−2.2 (2)
C13—C8—C9—C10	−1.2 (2)	B1—C20—C25—C24	−176.95 (14)
B1—C8—C9—C10	−174.43 (13)	C20—B1—C26—C27	−32.58 (18)
C8—C9—C10—C11	1.3 (2)	C14—B1—C26—C27	90.33 (15)
C9—C10—C11—C12	0.1 (2)	C8—B1—C26—C27	−148.27 (13)
C10—C11—C12—C13	−1.5 (2)	C20—B1—C26—C31	152.20 (13)
C11—C12—C13—C8	1.5 (2)	C14—B1—C26—C31	−84.89 (15)
C9—C8—C13—C12	−0.2 (2)	C8—B1—C26—C31	36.51 (18)
B1—C8—C13—C12	173.10 (13)	C31—C26—C27—C28	−0.8 (2)
C20—B1—C14—C19	30.90 (19)	B1—C26—C27—C28	−176.32 (14)
C8—B1—C14—C19	147.85 (13)	C26—C27—C28—C29	−0.3 (2)
C26—B1—C14—C19	−91.11 (16)	C27—C28—C29—C30	0.9 (2)
C20—B1—C14—C15	−155.60 (13)	C28—C29—C30—C31	−0.4 (2)
C8—B1—C14—C15	−38.64 (18)	C29—C30—C31—C26	−0.7 (2)
C26—B1—C14—C15	82.40 (16)	C27—C26—C31—C30	1.3 (2)
C19—C14—C15—C16	−0.8 (2)	B1—C26—C31—C30	176.85 (14)
B1—C14—C15—C16	−174.84 (14)		

### Hydrogen-bond geometry (Å, º)

Cg1 and Cg2 are the centroids of the C14–C19 and C8–C13 rings, respectively.

**Table:**

*D*—H···*A*	*D*—H	H···*A*	*D*···*A*	*D*—H···*A*
C3—H3···*Cg*1^i^	0.95 (2)	2.55 (2)	3.493 (2)	172 (2)
C2—H2*C*···*Cg*2^ii^	0.98	2.65	3.399 (2)	133
C5*A*—H5*B*···*Cg*2	0.99	2.78	3.621 (2)	144
C5*B*—H5*D*···*Cg*2	0.99	2.62	3.542 (2)	154

Symmetry codes: (i) *x*+1/2, −*y*+1/2, *z*+1/2; (ii) *x*+1, *y*, *z*.
